# Which PHQ-9 Items Can Effectively Screen for Suicide? Machine Learning Approaches

**DOI:** 10.3390/ijerph18073339

**Published:** 2021-03-24

**Authors:** Sunhae Kim, Hye-Kyung Lee, Kounseok Lee

**Affiliations:** 1 Department of Psychiatry, Hanyang University Medical Center, Seoul 04763, Korea; sunhk0906@hanyang.ac.kr; 2 Department of Nursing, College of Nursing and Health, Kongju National University, Kognju 32588, Korea; hklee@kongju.ac.kr

**Keywords:** PHQ-9, suicide, screening, machine learning

## Abstract

(1) Background: The Patient Health Questionnaire-9 (PHQ-9) is a tool that screens patients for depression in primary care settings. In this study, we evaluated the efficacy of PHQ-9 in evaluating suicidal ideation (2) Methods: A total of 8760 completed questionnaires collected from college students were analyzed. The PHQ-9 was scored in combination with and evaluated against four categories (PHQ-2, PHQ-8, PHQ-9, and PHQ-10). Suicidal ideations were evaluated using the Mini-International Neuropsychiatric Interview suicidality module. Analyses used suicide ideation as the dependent variable, and machine learning (ML) algorithms, k-nearest neighbors, linear discriminant analysis (LDA), and random forest. (3) Results: Random forest application using the nine items of the PHQ-9 revealed an excellent area under the curve with a value of 0.841, with 94.3% accuracy. The positive and negative predictive values were 84.95% (95% CI = 76.03–91.52) and 95.54% (95% CI = 94.42–96.48), respectively. (4) Conclusion: This study confirmed that ML algorithms using PHQ-9 in the primary care field are reliably accurate in screening individuals with suicidal ideation.

## 1. Introduction

Most individuals who die by suicide have visited mental health services providers within one year before death (rates of contact average 32%, range = 16–46%) [1,2]. These medical visits are an opportunity for clinicians to identify and refer patients at risk of suicide; however, patients often talk about their physical problems without discussing suicidal ideations and plans unless asked directly [3,4]. It can be easy to overlook suicide risk assessments even when the risk is high if the patient denies a suicide attempt or refuses a psychiatric evaluation.

Because depression and suicide are often associated in primary care, clinicians use depression tests as a suicide risk detection tool. However, depression screening tests were not designed to evaluate suicide-related risk factors [5]. In addition, the cost-effectiveness of the suicide risk assessment scale as a first-line screening strategy is limited because of the relatively low prevalence of suicide among primary care patients [6,7].

The Patient Health Questionnaire (PHQ-9) is an effective screening tool for major depressive disorders and is a reliable and useful evaluation tool for moderate symptoms of depression in various clinical environments [8,9,10]. This tool has been verified for screening for depression in the general population and various groups outside the primary care environment [11,12].

Item 9, the final item of PHQ-9, is generally used in research to determine the existence of suicidal ideation [13,14,15]. Therefore, item 9 is sometimes referred to as the PHQ-9 suicide question, because it specifically evaluates the frequency of passive accidents due to death or self-harm over the preceding two weeks. It is widely used as a single scale for studies on the prevalence of suicidal ideas [13,14,16,17,18]. In addition, item 9 of PHQ-9 can be used as a predictor of suicidal death or risk even when depression severity is reduced [19]. Higher scores in question 9 are better at predicting suicide risk and mortality [20]. Additionally, PHQ-9 has been used to detect patients at risk of suicide in several studies [19,21,22,23,24]. The American Heart Association Science Advisory recommends that all patients who answer “yes” to item 9 of PHQ-9 be immediately assessed for suicide [25].

However, the predictive value of PHQ-9 item 9 on suicide risk remains unclear. Additionally, some studies have argued that item 9 responses yield a high false-positive ratio by evaluating both the thought of passive death and desire for self-harm in a single-response question [13,16,20,23,24,26]. Therefore, studies are being conducted on the predictive value of item 9 of PHQ-9 for suicide or depression [19,26].

Classifying and predicting individuals with high suicide risk using PHQ-9 will assist with suicide prevention and intervention. Item 9 is widely used as a suicide screening tool; however, no studies have assessed the efficacy of PHQ-9 as a suicide screening tool using machine learning (ML) techniques. Machine learning algorithms have been proposed to improve diagnostic and prognostic accuracy and determine treatment options [27]. Various machine learning studies are focused on suicide prediction [28,29,30] and machine learning analysis has an advantage in its accuracy and scalability compared to conventional statistical approaches [29].

These can investigate a wide range of complex associations between many potential factors to generate algorithms that optimize predictions and reliably predict risk [31,32,33]. This study sought to effectively distinguish individuals with suicidal ideations by applying the latest ML algorithms to the PHQ-9 results and reveal suicide risk prediction accuracy.

## 2. Materials and Methods

This study used a part of the questionnaire dataset conducted at Kongju National University from 2013 to 2015. Informed written consent was obtained from all participants. In total, 8760 responses (4354 males, 4406 females) were analyzed [34]. A total of 742 (8.5%) participants were classified as a suicide group, and the remainder were classified as a control group (8018, 91.5%). This study was approved by the Kongju National University Institutional Review Board (approval No. KNU2015-38).

The Patient Health Questionnaire (PHQ) is a self-report questionnaire designed to help diagnose and detect mental disorders commonly encountered in the primary clinical setting [35]. It consists of 9 questions to help diagnose a major depressive disorder. PHQ-2 is composed of two items, depressed mood and anhedonia, which are the core symptoms among the diagnostic criteria for major depressive disorders in DSM-IV. The items score how often these feelings have occurred in the last two weeks. In this study, four versions were classified according to the combined criteria of the questionnaire items. These were named PHQ-2 using the sum of the first two questions; PHQ-8, incorporating 8 questions and excluding question 9; and PHQ-9, incorporating up to 9 questions. The sum of up to the 10th question (‘how difficult have these problems made it for you’), which is the question about difficulties due to depression symptoms, is referred to as PHQ-10 (Appendix A).

The Mini-International Neuropsychiatric Interview (MINI) was developed in the US and Europe in 1998 for the diagnosis of axis I psychiatric disorders in DSM-IV and the International Classification of Diseases 10th edition (ICD-10). It is used as a structured interview tool in multicenter clinical studies and epidemiological investigations [36]. Suicide risk is evaluated with 6 questions. In this study, participants who responded positively to questions 1−3, which are related to suicidal ideation, were classified into the suicide ideations group. In this study, the standardized Korean version 5.0 was used [37].

Responses to PHQ-9 were input into the ML algorithms to assess which questions influenced actual suicidal ideations. The algorithms used were k-nearest neighbors classification (KNN), linear discriminant analysis (LDA), and random forest classification. The k-nearest neighbors (kNN) is a non-parametric classification method that is simple and effective in many cases [38]. The kNN algorithm collects existing classes and classifies new classes based on the comparison measure. It has been used as a non-parametric technique for pattern recognition and statistical estimation [39]. Linear discriminant analysis (LDA) determines the correlation between a categorical variable and an interrelated variable. In LDA, an entity of interest is presumed to belong to only one of the groups [40]. Random forest algorithm is the grouping of tree predictors, such that each tree is influenced by the values of a random vector experimented both individually and with the same dissemination for all trees in the forest. Random forests are an operational tool in estimation. Introducing the proper type of chance makes them precise classifiers and regressors. Furthermore, random forests analyze the data by considering the strength of discrete analysts and their associations [41].

The abovementioned machine learning (ML) models are based on critical features. ML algorithms are designed to maximize their clinical significance and generalization potential. In fact, ML models have been shown to be able to predict suicide tendencies [27]. Although ML algorithms have improved the accuracy of suicide risk detection; for a number of reasons, perfect predictions using ML are not possible and result in false positives and false positives [31].

Therefore, clinical implications are important to reduce these errors and increase accuracy. We analyzed three machine learning algorithms accordingly. The target variable (y) was binary. We collected 8713 questionnaires, from which 5576 responses were used as training data, 1395 were used as validation data, and 1742 were used as test data. In LDA, 6971 people were used as train data and 1742 were used as test data. Suicide ideation with/without group ML analyses were performed using JASP v0.14.4 (Amsterdam, Netherland). The dependent variable was suicidal ideation and the independent variables were combinations of 10 items of PHQ-9. A Chi-Square test of independence and a t-test were conducted. In addition, we calculated the sensitivity, specificity, positive predictive value (PPV), and negative predictive value (NPV) with a 95% confidence interval (95% CI) by calculating the sum according to each version of PHQ-2, PHQ-8, PHQ-9, and PHQ-10. The calculation consisted of suicide ideation groups with a PHQ-2 cutoff score > 1, PHQ-8 cutoff score > 4, PHQ-9 cutoff score > 5, PHQ-10 cutoff score > 5.

The area under the curve (AUC) of the receiver operating curve (ROC) was measured for each algorithm. An AUC close to 1 indicates a better model. An AUC of 0.5–0.6 indicates a coincidence and 0.6–0.7 indicates a bad model, while 0.7–0.8 is worthless, 0.8–0.9 is good, and 0.9–1.0 is excellent [42]. All statistical analyses were performed using JASP v0.14.4 (Amsterdam, the Netherland) and MedCalc v19.6.1 (MedCalc Software, Mariakerke, Belgium). All *p* values were obtained using two-tailed tests, and *p* < 0.05 was considered statistically significant. To control for multiple comparisons, a Bonferroni corrected α was adopted for each item’s score (0.05/10 = 0.005).

This study aimed to differentiate the suicide ideation group using a patient health questionnaire (PHQ)-9 items by applying the latest ML algorithm models. Validation was performed using MINI as the gold standard. We used the subtotal score of PHQ-9 items, sensitivity, specificity, positive predictive value (PPV), negative predictive value (NPV), and area under the receiver operating characteristic curve (AUC) for comparison with the clinical diagnosis of suicide ideation using 95% confidence intervals for each measure [43].

## 3. Results

### 3.1. Experimental Results

#### 3.1.1. General Characteristics

Of all participants, 742 (8.5%) were included in the suicidal ideation group. In the suicidal ideation group, the proportion of women was significantly higher than men (X^2^ = 48.61, *p* < 0.001, Table 1). The age difference between groups was not significant; however, the total score for each PHQ-9 item and the sum of each scoring method was significantly higher in the suicidal ideation group (Table 1).

#### 3.1.2. PHQ Cutoff Scores

When 5 points were used as a cut-off point in the evaluation using the total score of PHQ-9, the sensitivity and specificity were 71.56% and 76.54%. The AUC was 0.817 (Table 2); however, the PPV was 22.01%. Interestingly, the PPV was low (17.9–22.01%) in all four types of evaluation using the total score. In contrast, the NPV was high (96.68–97.10%). The cut-off threshold score was 5 for the sensitivity and specificity of PHQ-9 (PPV = 22.01, NPV = 96.68).

#### 3.1.3. PHQ Item Combination by ML Technique

The ML analysis revealed an accuracy of >90%, which was excellent in all item combinations. An accuracy of 94.4% was found in the PHQ-10 (Table 3). The AUC was 0.848 in ML with the linear discriminant technique in PHQ-8. When nine items were used, the positive and negative predictive values were 84.95% (95% CI = 76.03–91.52) and 95.54% (95% CI = 94.42–96.48), respectively, which showed good diagnostic performance.

## 4. Discussion

The purpose of this study was to predict and report suicidal ideations using the ML analyses of a combination of 10 items from the PHQ-9, which is the most used depression assessment tool in primary healthcare. We found differences between ML techniques; however, we confirmed that KNN, LDA, and random forest all predicted suicidal ideations at an excellent level. In particular, all techniques showed a high level of accuracy. This means that the depression screening tool can be used to predict suicide risk using ML without requiring direct confirmation of suicide ideations and attempts.

The ninth item on the PHQ-9 is used to indicate the existence of suicidal ideations. In fact, it is widely used as a single measure of the prevalence of suicidal ideations [13,14,15,16,17,18]. All ML techniques showed that PHQ-9 and PHQ-10 scored a higher accuracy than PHQ-2 and 8, which exclude item 9. The AUC analysis revealed that LDA and random forest analyses were superior (0.816–0.846). In addition, PPV and NPV were 62.71–84.95% and 93.82–95.54%, which were higher than the PPVs of PHQ-2 and 8 (37.14–69.57%). The ninth item asks patients “How often have you been bothered by thoughts that you would be better off dead or of hurting yourself in some way?” This question refers to self-harm and suicidal thoughts. Most people with suicidal ideations or self-harm respond positively to this question [16,44]. In our study, the predicted values of PHQ-9 and PHQ-10—which included this item—were more related to suicide. Therefore, all PHQ-9 items using ML, including item 9, better predict suicidal ideation than the shortened PHQ-2 and PHQ-8.

There are limitations to the single use of the ninth item of PHQ-9 as an effective suicide screening tool. Further studies are assessing which items are the best predictors of suicide risk [16,19,45,46,47]. In this study, PHQ-9 and PHQ-10 were more accurate than PHQ-2 and PHQ-8 even when using a cut-off point on the total number of PHQ questions, and without using ML. This indicated that the inclusion of PHQ-9 item 9 can predict suicidal ideations even while controlling for different statistical techniques. Our study showed similar results to a previous study that found that having positive scores on the depressive symptoms in items 1–8 was less important than item 9 when assessing suicide [19,20]. The PHQ-9 is designed to measure the severity of depression, but if it was specifically focused on suicidal ideation, its prediction of suicidal ideation might be more accurate. In particular, since this study has confirmed the possibility of predicting suicide risk through PHQ-9—including item 9, which measures suicidal ideation— suicide risk can be considered to be an indirect measurement compared with other suicide screening tools. Rather than directly assessing suicidal ideation, indirect measures such as an emotion regulation questionnaire and an anger rumination scale are better predictors of actual suicide attempts [30]. Therefore, PHQ-9— a tool commonly used in clinical settings or various groups— would be more useful and effective in discriminating thoughts about suicide indirectly than a measurement tool that directly screens for suicide in an early screening measure. A previous study sought to predict suicide risk using the Columbia-Suicide Severity Rating Scale (C-SSRS), based on the PHQ-9 cut-off value. They showed high sensitivity and specificity, but low PPV (22.0–30.4%) [45]. In this study, measurements using the cut-off value of the existing statistical method showed similar PPV values (17.90–22.01%); however, the PHQ-9 had the highest PPV (84.95%) score when using the ML technique. Because the PHQ-9 was originally developed to screen for depression and not for suicide, these results indicate that PHQ-9 and PHQ-10 would be very useful for screening suicide risk in a primary care situation.

The accurate prediction of suicide attempts requires a complex combination of hundreds of risk factors; therefore, traditional statistical techniques are not ideal for this analysis. For example, generally strong predictors—such as previous suicidal behavior, depression, despair, or male gender—also appear to be weak predictors of risk [46,47,48]. A recent meta-analysis has reported that predicting suicide attempts using previously known risk factors is not effective due to methodological limitations (AUC = 0.56–0.58). They argue that it is necessary to use a risk algorithm using ML to improve this [49]. These techniques will accurately predict suicidal behavior and actual suicide through longitudinal records of patients [31,33,34], socio-demographic information, psychopathological factors, and control for risk factors [49]. Other ML studies have shown suicidal ideation as a better potential predictor of suicidal risk than suicidal attempts [50]. Suicide ideation is considered to be an important precursor to a later attempted and completed suicide and can be considered a first step on the pathway to suicide [51]. Therefore, the strength of this study is that it analyzed the usefulness of PHQ-9 as a suicide ideation screening tool for the first steps leading to suicide attempts and planning through ML algorithms. Screening tools should be applied differently to determine whether actionable risks exist and identify risk factors that should be ignored. This will enable easy management by front-line staff. Furthermore, it should be highly sensitive to confidently rule out patients who do not show any visible risk [52]. Hence, the PHQ-9 is suitable as a screening tool in the primary care setting, which has been confirmed through this study. However, these algorithmic predictions cannot predict when these people will manifest suicidal risk. Further research is required to assess the correct therapeutic approach for those at risk [29].

There are limitations to this study. First, it is difficult to say that these results are representative of the entire population, as the survey was conducted at one university. Second, as a self-reported study, there is an inherent respondent bias. This survey was conducted for a large number of students, but it was conducted only in the form of a self-report, and we could only use the MINI model of suicidality for comparison. Third, there was no information on clinical diagnosis or psychiatric treatment because this was a study in a non-clinical group. This is a retrospective analysis using existing data; therefore, it was difficult to obtain additional information. Fourth, the MINI suicidality module has not been sufficiently verified. However, the most effective screening method for evaluating suicide is to ask directly and the first three questions of MINI would not have been efficient in evaluating suicidal ideations. Although in this study we have not considered the effect of depression, it may act as a confounding variable. Therefore, future confirmatory studies will need to be conducted on individuals with major depressive disorder.

Nevertheless, this study confirmed that analysis using ML can be used very accurately when screening for people with suicidal ideations using PHQ-9, a simple depression screening tool. The possibility of predicting and evaluating the risk using national prevalence studies or simple evaluation in the primary care field was presented. Justification for population-based screening and prevention would require both evidence that the screening predicts suicidal risk in the general population and evidence that some feasible intervention can reduce that risk [53,54]. Therefore, in the future, if clinical data are added and analyzed through long-term follow-up for a wider range of groups, the value of PHQ-9 in suicide risk assessment will increase.

## 5. Conclusions

This study confirmed that ML with PHQ-9 provides reliable accuracy in classifying and predicting suicidal ideations of individuals. All ML algorithms indicated that if the PHQ-9 included item 9, then the prediction accuracy was high. AUC analysis revealed that LDA and random forest analyses were superior among all ML algorithms tested. These findings can help clinicians to detect and treat high-risk suicide groups early in the primary care field.

## Figures and Tables

**Table 1:** Differences in response and general characteristics of each group according to suicidal ideation.

Variable	Dimension	Overall (*n* = 8761)	Groups Based on Suicidal Ideation		X^2^ or t	*p*
			No Suicidal Ideation (*n* = 8019, 91.5%)	With Suicidal Ideation (*n* = 742, 8.5%)		
sex	male	4354 (49.7%)	4073 (50.8%)	278 (37.5%)	48.61	<0.001
age		18.25 ± 8.64	18.25 ± 9.01	18.25 ± 2.41	0.01	0.996
each item’s score	item1	0.54 ± 0.64	0.47 ± 0.59	1.25 ± 0.78	26.35	<0.001
	item2	0.59 ± 0.67	0.54 ± 0.63	1.16 ± 0.80	24.90	<0.001
	item3	0.79 ± 0.85	0.75 ± 0.82	1.26 ± 0.98	13.55	<0.001
	item4	0.63 ± 0.78	0.59 ± 0.75	1.09 ± 0.97	16.77	<0.001
	item5	0.13 ± 0.42	0.11 ± 0.37	0.43 ± 0.71	12.17	<0.001
	item6	0.86 ±0.79	0.81 ± 0.76	1.43 ± 0.89	18.19	<0.001
	item7	0.38 ± 0.66	0.32 ± 0.58	1.09 ± 0.93	22.26	<0.001
	item8	0.15 ± 0.43	0.12 ± 0.38	0.48 ± 0.74	13.10	<0.001
	item9	0.03 ± 0.28	0.01 ± 0.12	0.55 ± 0.72	20.13	<0.001
	item10	0.25 ± 0.51	0.21 ± 0.45	0.73 ± 0.77	27.61	<0.001
subtotal score	PHQ-2	1.12 ± 1.16	1.00 ± 1.06	2.40 ± 1.41	26.42	<0.001
	PHQ-9	4.12 ± 3.62	3.70 ± 3.17	8.69 ± 4.86	27.44	<0.001
	PHQ-8	4.06 ± 3.50	3.68 ± 3.14	8.14 ± 4.48	26.51	<0.001
	PHQ-10	4.36 ± 3.90	3.89 ± 3.39	9.40 ± 5.27	27.98	<0.001
MINI suicidality	total	0.69 ± 2.86	0.17 ± 0.82	6.39 ± 7.32	23.16	<0.001

Values were presented as mean ± SD or *n* (%).

**Table 2:** Diagnostic characteristics of suicidal ideation assessment using PHQ cutoff scores.

Variable	Cutoff	Accuracy	AUC	Sensitivity (%)	Specificity (%)	PPV (%)	NPV (%)
PHQ-2	>1	0.680	0.786	77.36 (74.17–80.32)	67.16 (66.12–68.19)	17.90 (17.18–18.64)	96.97 (96.56–97.34)
PHQ-8	>4	0.682	0.803	78.30 (75.16–81.22)	67.29 (66.25–68.31)	18.13 (17.41–18.88)	97.10 (96.69–97.47)
PHQ-9	>5	0.761	0.817	71.56 (68.17–74.79)	76.54 (75.60–77.46)	22.01 (21.00–23.07)	96.68 (96.29–97.03)
PHQ-10	>5	0.744	0.824	75.20 (71.93–78.27)	74.31 (73.34–75.26)	21.31 (20.40–22.26)	97.00 (96.62–97.35)

AUC: area under the receiver operative characteristic curve; PPV: positive predictive value; NPV: negative predictive value.

**Table 3:** Diagnostic characteristics of suicidal ideation assessment using PHQ Item combination by ML technique.

Variable	ML Methods	Validation Accuracy	Test Accuracy	OOB Accuracy	AUC	Sensitivity (%)	Specificity (%)	PPV (%)	NPV (%)
PHQ-2	KNN	0.918	0.907	n/a	0.700	4.97 (2.17–9.56)	99.43 (98.92–99.74)	47.06 (22.98–72.19)	91.13 (89.69–92.43)
	Linear Discriminant	n/a	0.916	n/a	0.803	30.46 (23.24–38.47)	97.36 (96.45–98.09)	52.27 (41.35–63.04)	93.65 (92.37–94.78)
	random forest	0.924	0.920	0.070	0.625	10.81 (6.31–16.96)	99.56 (99.1–99.82)	69.57 (47.08–86.79)	92.32 (90.96–93.54)
PHQ-8	KNN	0.921	0.920	n/a	0.738	16.18 (10.42–23.46)	98.43 (97.69–98.98)	46.81 (32.11–61.92)	93.22 (91.91–94.37)
	Linear Discriminant	n/a	0.918	n/a	0.848	34.53 (26.68–43.06)	96.79 (95.8–97.6)	48.48 (38.32–58.75)	94.41 (93.19–95.48)
	random forest	0.913	0.920	0.131	0.768	9.56 (5.19–15.79)	98.62 (97.92–99.13)	37.14 (21.47–55.08)	92.73 (91.39–93.93)
PHQ-9	KNN	0.931	0.932	n/a	0.736	31.29 (23.91–39.45)	98.99 (98.36–99.42)	74.19 (61.5–84.47)	93.94 (92.68–95.04)
	Linear Discriminant	n/a	0.936	n/a	0.816	41.61 (33.91–49.64)	98.92 (98.27–99.37)	79.76 (69.59–87.75)	94.28 (93.05–95.36)
	random forest	0.951	0.943	0.409	0.841	51.97 (43.73–60.14)	99.11 (98.51–99.51)	84.95 (76.03–91.52)	95.54 (94.42–96.48)
PHQ-10	KNN	0.926	0.927	n/a	0.750	27.82 (20.4–36.25)	98.51 (97.76–99.07)	62.71 (49.15–74.96)	93.82 (92.51–94.97)
	Linear Discriminant	n/a	0.941	n/a	0.846	43.18 (34.59–52.08)	98.65 (97.92–99.17)	74.03 (62.77–83.36)	95.12 (93.92–96.14)
	random forest	0.936	0.944	0.424	0.843	44.03 (35.47–52.86)	98.99 (98.33–99.43)	79.73 (68.78–88.19)	95.13 (93.93–96.15)

AUC: area under the receiver operative characteristic curve; PPV: positive predictive value; NPV: negative predictive value; n/a: not applicable.

**Figure A1:**
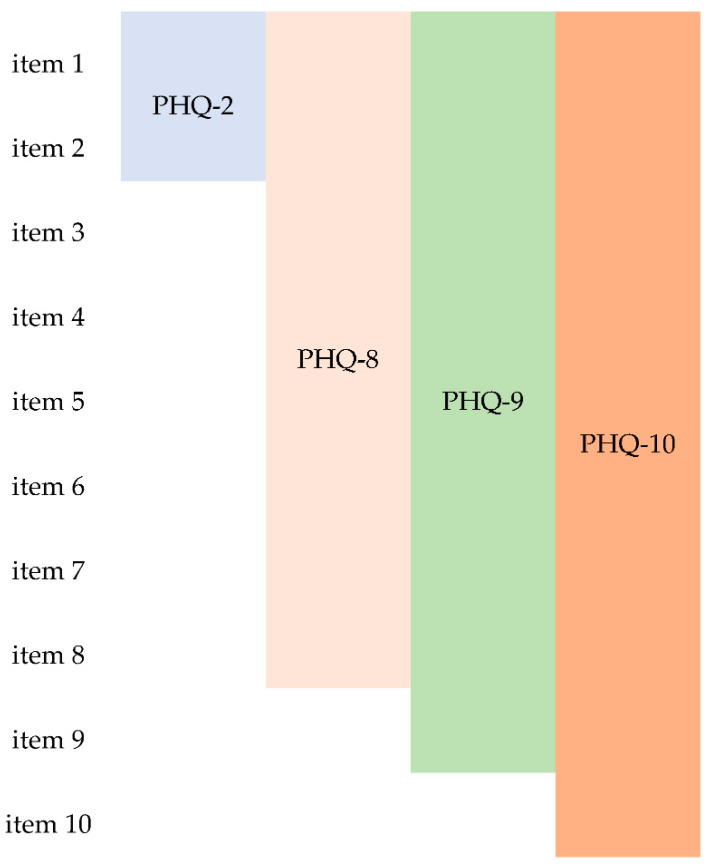
Independent variable inputted according to PHQ combinations.

## Data Availability

The data presented in this study are available on request from the corresponding author. The data are not publicly available due to ethical restrictions.

## Appendix A

**Table A1:** PHQ-9 items.

No.	Question
item 1	Little interest or pleasure in doing things
item 2	Feeling down, depressed, or hopeless
item 3	Trouble falling/staying asleep or sleeping too much
item 4	Feeling tired or having little energy
item 5	Poor appetite or overeating
item 6	Feeling bad about yourself—or that you are a failure or have let yourself or your family down
item 7	Trouble concentrating on things, such as reading the newspaper or watching television
item 8	Moving or speaking so slowly that other people could have noticed? Or the opposite—being so fidgety or restless that you have been moving around a lot more than usual
item 9	Thoughts that you would be better off dead or of hurting yourself in someway
item 10	If you checked off any problems, how difficult have these problems made it for you to do your work, take care of things at home, or get along with other people?
